# Hybrid Operating Room Applications in Otolaryngology: A Seven-Year Single-Center Experience with Image-Guided and Multidisciplinary Procedures

**DOI:** 10.3390/diagnostics16142273

**Published:** 2026-07-21

**Authors:** Tzu-Chen Huang, Hao-Chun Hung, Shu-Wei Yeh, Chang-Yo Pan, Mei-Wen Nian, Iva Lin, Chung-Hsiung Chen, Stella Chin-Shaw Tsai

**Affiliations:** 1 Department of Otolaryngology, Tungs’ Taichung MetroHarbor Hospital, Taichung 435403, Taiwan; victor8357307@gmail.com (T.-C.H.); anke801211@gmail.com (M.-W.N.); 2 Department of Medical Imaging, Tungs’ Taichung MetroHarbor Hospital, Taichung 435403, Taiwan; akina6827@gmail.com; 3 Department of Medical Education, Tungs’ Taichung MetroHarbor Hospital, Taichung 435403, Taiwan; ericyeh0224@gmail.com (S.-W.Y.); 107001981@365.cmu.edu.tw (C.-Y.P.); 4 Department of Medical Research, Tungs’ Taichung MetroHarbor Hospital, Taichung 435403, Taiwan; 5 Teachers College, Columbia University, New York, NY 10027, USA; il2527@tc.columbia.edu; 6 Department of Post-Baccalaureate Medicine, National Chung Hsing University, Taichung 402202, Taiwan

**Keywords:** hybrid operating room, otolaryngology, intraoperative imaging, image-guided surgery, endovascular intervention, multidisciplinary care

## Abstract

**Background/Objectives**: Hybrid operating rooms combine advanced intraoperative imaging, endovascular capabilities, and multidisciplinary resources within a single procedural environment. However, their use in otolaryngology remains insufficiently characterized. This study evaluated institutional patterns of hybrid operating room use, principal clinical indications, multidisciplinary involvement, and perioperative resource utilization in otolaryngology. **Methods**: We conducted a retrospective single-center study of eligible otolaryngologic procedures performed between 1 October 2018 and 31 December 2025. Patient characteristics, operative sites, hybrid operating room applications, multidisciplinary involvement, intraoperative blood loss, postoperative intensive care unit admission, and length of hospital stay were analyzed. **Results**: A total of 55 unique procedures were included. The median age was 46.0 years (interquartile range, 33.0–58.5 years; range, 5–76 years), and 33 patients (60.0%) were male. Computed tomography-based localization and navigation represented the predominant application, accounting for nearly three-quarters of procedures. The sinonasal cavity, nasopharynx, and skull base were the most frequently treated anatomical regions, comprising approximately 60% of operative sites. Angiography and endovascular intervention constituted the second most common application. Multidisciplinary collaboration, most frequently involving cardiovascular surgery and interventional radiology, was required in nearly one-quarter of procedures. Procedures relying primarily on intraoperative imaging were associated with a median estimated blood loss of 20 mL and a median hospital stay of 3 days. Cases requiring vascular, cardiopulmonary, or other advanced hybrid capabilities showed greater postoperative resource utilization, including more frequent intensive care admission and longer hospitalization. **Conclusions**: The hybrid operating room served as a versatile platform for image-guided, vascular, and multidisciplinary procedures in otolaryngology. Its capabilities were used during the management of anatomically complex and high-acuity cases, while differences in postoperative resource utilization appeared to reflect procedural complexity and baseline clinical risk.

## 1. Introduction

The hybrid operating room integrates intraoperative imaging, navigation, and interventional capabilities within a single operative environment. Its application has increasingly expanded into otolaryngology, allowing diagnostic and therapeutic procedures to be performed during the same anesthetic session without relocating the patient [1].

The complex anatomy of the head and neck region presents unique spatial challenges that require robust imaging solutions. Accurate evaluation of sinonasal structures and the skull base is fundamental to safe surgical practice [2]. Real-time intraoperative computed tomography and integrated navigation systems may provide valuable support, particularly when delineating tumor margins or navigating complex sinonasal and skull-base anatomy [3]. These tools may assist anatomical localization and enable confirmation of the intended procedural result before the patient leaves the operating room.

Beyond structural navigation, otolaryngology practice frequently involves highly vascular pathologies and high-acuity scenarios requiring cross-disciplinary expertise. Advanced vascular imaging and immediate embolization are critical components in the surgical management of vascular-rich lesions, such as advanced juvenile nasopharyngeal angiofibroma [4]. An integrated angiographic suite may also provide immediate access to endovascular intervention in the event of vascular complications during complex skull-base surgery [5]. Combining open surgical approaches with immediate endovascular capabilities in one location mitigates the severe risks associated with intraoperative patient transport and delayed interventions.

Despite these potential applications, evidence describing hybrid operating room utilization across otolaryngology remains limited. Previous reports have mainly focused on individual procedures, selected technologies, or small case series, and little is known about longitudinal institutional patterns of use, principal clinical indications, multidisciplinary participation, and perioperative resource requirements. Therefore, this retrospective study evaluated the evolving institutional utilization, principal clinical applications, multidisciplinary involvement, and perioperative resource requirements of otolaryngology procedures performed in a hybrid operating room over a seven-year period.

## 2. Materials and Methods

### 2.1. Study Design, Data Extraction, and Patient Selection

**Figure 1:**
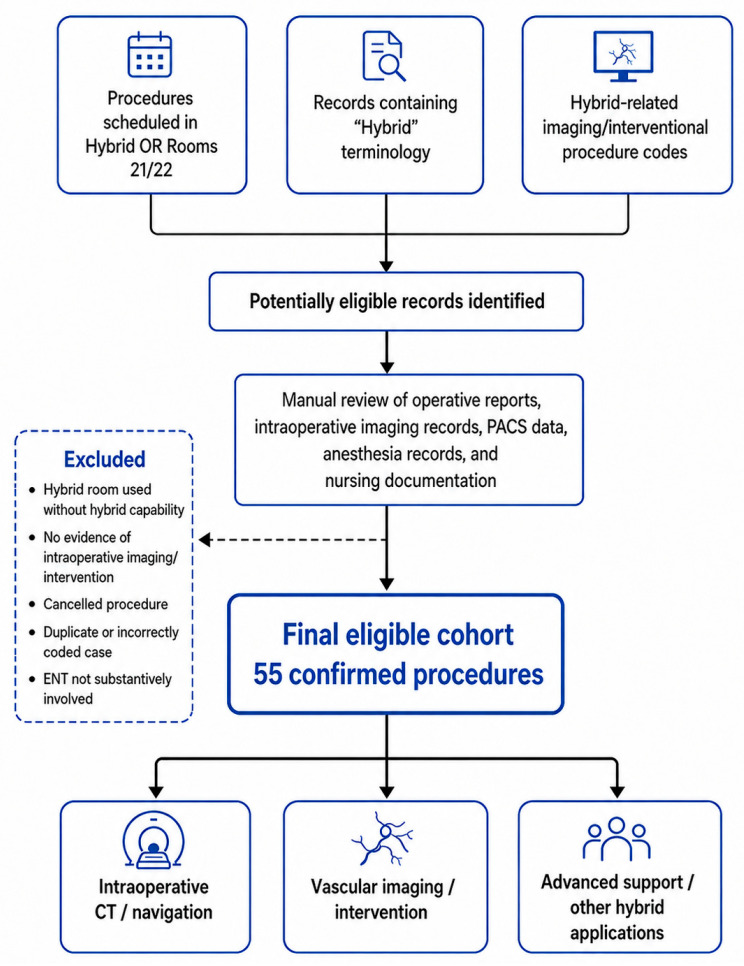
Study flowchart for identifying eligible otolaryngology hybrid operating room procedures.

A retrospective institutional review was conducted to identify and evaluate otolaryngology procedures performed within the hybrid operating room over the study period from 1 October 2018 to 31 December 2025. This study used a complete retrospective census rather than random or convenience sampling. All potentially eligible otolaryngology procedures performed in the hybrid operating room during the defined study period were screened, and all procedures meeting the prespecified eligibility criteria were included. Following the installation of the hybrid operating room at our institution in 2018, a structured pathway was used to identify and verify eligible procedures, as detailed in Figure 1. Potentially eligible medical records were initially captured through three distinct avenues to ensure a comprehensive sample. These search strategies included identifying procedures specifically scheduled in designated hybrid operating rooms, querying medical records for clinical narratives containing hybrid-related terminology, and extracting cases associated with hybrid-specific imaging or interventional procedure codes. Following this initial broad identification, a manual review of all source records was systematically performed to confirm true eligibility. This verification process encompassed the evaluation of operative reports, intraoperative imaging records, picture archiving and communication system (PACS) data, anesthesia records, and nursing documentation. Procedures were rigorously evaluated and subsequently excluded if the hybrid suite was utilized merely as a standard operating theater without the actual application of its integrated imaging, interventional, or advanced support capabilities. Additional predefined exclusion criteria involved cases lacking documented evidence of intraoperative imaging or intervention, cancelled procedures, duplicate or incorrectly coded records, and surgeries in which the otolaryngology service was not substantively involved. Upon applying these rigorous criteria, a final eligible cohort of 55 confirmed unique procedures was established for subsequent analysis.

Selection for the hybrid operating room was determined during preoperative planning according to the anticipated need for intraoperative computed tomography or navigation, immediate procedural verification, angiographic or endovascular intervention, or advanced cardiopulmonary support. Decisions were made on a case-by-case basis by the treating otolaryngology team, with multidisciplinary consultation when required. No uniform allocation protocol was applied throughout the entire study period.

### 2.2. Hybrid Operating Room Environment and Procedural Setup

The hybrid operating room integrates high-resolution intraoperative imaging, optical navigation, and advanced interventional capabilities within a single operative space [6]. At our institution, the hybrid operating room was equipped with both a floor-mounted robotic angiography system (ARTIS Pheno; Siemens Healthineers, Erlangen, Germany) and a sliding computed tomography gantry (Siemens Healthineers, Erlangen, Germany). The robotic C-arm provided high-resolution fluoroscopy, digital subtraction angiography, and rotational three-dimensional imaging, whereas the sliding computed tomography system enabled acquisition of conventional cross-sectional images within the operative suite without transferring the anesthetized patient to a separate radiology department. Together, these systems supported immediate anatomical assessment, lesion localization, navigation registration, vascular evaluation, and post-procedural verification during the same anesthetic session. The operating room also incorporated a high-definition endoscopic imaging system (KARL STORZ SE & Co. KG, Tuttlingen, Germany) and, when indicated, a robot-assisted surgical platform (da Vinci Surgical System; Intuitive Surgical, Sunnyvale, CA, USA).

**Figure 2:**
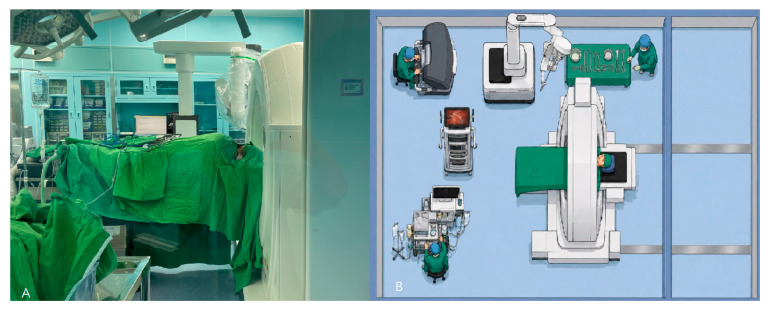
Hybrid operating room environment and intraoperative imaging setup: (**A**) Intraoperative deployment of the robotic imaging system, enabling fluoroscopy, digital subtraction angiography, and rotational three-dimensional imaging during surgery. (**B**) Schematic representation of the integrated hybrid operating room configuration, showing the spatial relationship among the operative table, imaging gantry, da Vinci Single Port system, and surgical team. This arrangement permits intraoperative imaging, navigation, robot-assisted surgery, and multidisciplinary intervention within the same procedural environment.

Representative intraoperative deployment of the imaging system and the operating room configuration are shown in Figure 2. By incorporating a robotic C-arm or sliding computed tomography gantry, the surgical suite enables surgeons to perform immediate anatomical assessments without necessitating high-risk patient transport. The typical procedural workflow in this specialized environment begins with standard patient positioning and registration using navigation arrays. This preparatory phase is seamlessly followed by the deployment of the intraoperative imaging system to acquire precise cone-beam computed tomography scans. These resulting scans are then directly imported into the surgical navigation workstation to facilitate the precise localization of critical neurovascular structures, complex tumor margins, or obscure sinonasal boundaries [7]. Furthermore, the room is equipped with an integrated angiographic suite. This inclusion permits immediate transitions from open surgical resection to endovascular interventions, such as selective angiography or embolization, should high-acuity vascular scenarios arise [8]. Advanced cardiopulmonary support, including extracorporeal membrane oxygenation, was available when required for selected high-risk airway procedures. Ultimately, this multifaceted setup allows diagnostic imaging, definitive surgical resection, immediate procedural verification, and multidisciplinary interventions to be executed concurrently and efficiently.

### 2.3. Clinical Variables and Data Collection

For each eligible procedure, demographic, clinical, procedural, and perioperative data were extracted from the electronic medical record. Demographic variables included age, sex, and documented comorbidities. Clinical variables included the primary diagnosis, anatomical site, prior treatment history, and whether the procedure involved robot-assisted surgery or multidisciplinary participation. Procedural variables included the type of hybrid operating room modality used, the principal clinical application, anesthesia duration, operative duration, estimated blood loss, postoperative intensive care unit admission, duration of intensive care unit stay, postoperative intubation, nasogastric tube use, and total hospital length of stay.

Anesthesia duration was defined as the interval from anesthesia induction to completion of anesthesia care. Operative duration was defined as the interval from skin incision or initiation of the principal surgical procedure to completion of surgery. Intensive care unit and hospital lengths of stay were calculated from the corresponding admission and discharge dates. When discrepancies were identified among source records, the operative report and anesthesia record were considered the primary sources for procedural timing, whereas discharge documentation was used for hospitalization outcomes.

For procedures involving angiography or endovascular intervention, the clinical intent was categorized from the operative and angiographic records as either preoperatively planned and integrated into the operative session or unplanned emergency/rescue management.

### 2.4. Classification of Hybrid Operating Room Applications

Each procedure was assigned a single principal hybrid operating-room application to avoid double-counting. The predefined categories were (1) computed tomography localization or navigation, (2) immediate procedural verification, (3) angiography or endovascular intervention, and (4) advanced cardiopulmonary support. Computed tomography localization or navigation included procedures in which intraoperative imaging was used to localize lesions, define anatomical relationships, guide surgical navigation, or identify difficult anatomical targets. Immediate procedural verification referred to repeat imaging performed after the principal intervention to confirm lesion removal, hardware removal, or completion of the intended surgical result. Angiography or endovascular intervention included selective angiography, embolization, balloon-assisted procedures, carotid stenting, and covered-stent placement. Advanced cardiopulmonary support referred to procedures performed using extracorporeal membrane oxygenation.

When more than one hybrid operating room capability was used during the same procedure, the principal application was assigned according to the primary clinical objective documented in the operative report. Procedures were classified as angiography or endovascular intervention when vascular assessment, protection, embolization, or rescue treatment was the principal purpose, even if intraoperative computed tomography or robot-assisted surgery was also used. Otherwise, imaging-based procedures were classified according to whether imaging was used primarily for localization/navigation or for immediate procedural verification. Principal application categories were mutually exclusive. In contrast, modality-based analyses reflected all technologies used during the procedure and were not necessarily mutually exclusive; therefore, a single procedure could contribute to more than one modality-based subgroup. Robot-assisted surgery was recorded separately as an overlapping modality and was not considered a principal application category.

### 2.5. Multidisciplinary Involvement

Multidisciplinary participation was defined as direct procedural involvement by at least one specialty in addition to otolaryngology–head and neck surgery during the same operative session. Participating specialties included cardiovascular surgery, interventional radiology, plastic and reconstructive surgery, neurosurgery, thoracic surgery, and the extracorporeal membrane oxygenation team. Consultation without direct procedural participation was not classified as multidisciplinary involvement.

### 2.6. Study Outcomes

The primary study outcome was the distribution of principal hybrid operating room applications across the study period. Secondary outcomes included annual and cumulative procedural volume, anatomical distribution, multidisciplinary participation, and perioperative characteristics according to hybrid operating room modality. Perioperative outcomes included anesthesia duration, operative duration, estimated blood loss, postoperative intensive care unit admission, intensive care unit length of stay, postoperative intubation, nasogastric tube use, and total hospital length of stay.

As the study was descriptive and included clinically heterogeneous procedures, no causal comparisons among hybrid operating room modalities were prespecified. The single extracorporeal membrane oxygenation-supported procedure was presented descriptively and was not included in formal comparative testing.

### 2.7. Data Verification and Quality Control

Data extraction was performed using a structured case-review form. Basic demographic and hospitalization data were initially extracted by a postgraduate-year physician, while procedural details and hybrid operating room modality were reviewed by an otolaryngology resident. Eligibility, multidisciplinary participation, and application classification were subsequently verified by an attending otolaryngologist. Ambiguous cases and discrepancies were adjudicated by the principal investigator.

Duplicate cases were identified using the medical record number and procedure date. The previously published cases described in the Oral Oncology letter were retained in the present longitudinal cohort to preserve the complete institutional experience, but were identified as previously reported cases and counted only once [1]. The present study addressed a distinct research question focused on institutional utilization, procedural categories, multidisciplinary integration, and perioperative characteristics.

### 2.8. Statistical Analysis

Continuous variables were assessed for distribution and are presented as median and interquartile range because most perioperative variables were non-normally distributed. Categorical variables are presented as numbers and percentages. Annual case numbers were calculated by year of surgery, and cumulative case accrual was displayed graphically. Analyses were primarily descriptive owing to the clinical heterogeneity and small size of several modality-based subgroups, particularly the extracorporeal membrane oxygenation group. Counts from overlapping modality-based subgroups were not summed to derive the overall cohort size. The temporal association between calendar year and annual procedural volume was assessed using Spearman’s rank correlation coefficient. Statistical analyses were performed using SPSS Statistics version 28.0 (IBM Corp., Armonk, NY, USA).

### 2.9. Ethical Considerations

The study was approved by the Institutional Review Board of Tungs’ Taichung MetroHarbor Hospital (approval no. 115010-T). The requirement for informed consent was waived due to the retrospective study design and the use of de-identified clinical data. The study was conducted in accordance with the Declaration of Helsinki. All clinical images used for publication were de-identified before analysis and figure preparation.

## 3. Results

### 3.1. Patient and Procedural Characteristics

A total of 55 unique otolaryngology procedures performed in the hybrid operating room were included in the final cohort. The median patient age was 46.0 years (interquartile range, 33.0–58.5 years; range, 5–76 years), and 33 patients (60.0%) were male. The most common primary anatomical site was the sinonasal cavity, nasopharynx, or skull base, accounting for 33 procedures (60.0%). Among these 33 procedures, 30 (90.9%) were endoscopic sinus procedures performed for chronic rhinosinusitis. The remaining procedures comprised one transsphenoidal surgery for a benign pituitary adenoma, one procedure for recurrent nasopharyngeal carcinoma, and one procedure for chordoma. This was followed by the neck or thyroid in 10 (18.2%) procedures, the oral cavity or mandible in 5 (9.1%), the oropharynx or larynx in 4 (7.3%), and the salivary glands in 3 (5.5%).

Robot-assisted surgery was performed in 4 cases (7.3%), while 13 procedures (23.6%) involved direct multidisciplinary participation. Comorbidity data were available for 47 procedures. Hypertension was the most frequently documented comorbidity, occurring in 9 patients (19.1%), followed by diabetes mellitus in 4 (8.5%), cardiovascular or cerebrovascular disease in 2 (4.3%), hyperlipidemia in 2 (4.3%), and gout in 3 (6.4%). Chronic hepatitis and asthma were each documented in 1 patient (2.1%). Detailed demographic and procedural characteristics are summarized in Table 1.

### 3.2. Temporal Evolution of Hybrid Operating Room Utilization

**Figure 3:**
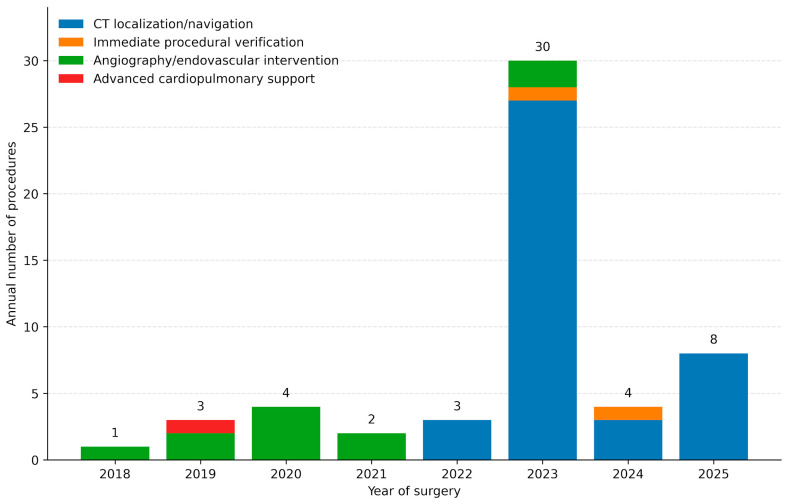
Annual number of otolaryngology hybrid operating room procedures according to principal application, 2018–2025. Each procedure was counted once according to its principal hybrid operating room application. The 2018 observation period began on 1 October 2018 and therefore represents a partial calendar year.

The cumulative number of otolaryngology hybrid operating room procedures increased from 2018 through 2025, reaching 55 unique procedures by the end of the study period (Figure 3). Annual procedural volume showed a positive association with calendar year (Spearman’s ρ = 0.735, *p* = 0.038). The largest annual increase occurred in 2023, primarily reflecting expanded use of intraoperative computed tomography for localization, navigation, and procedural verification. Early applications were predominantly vascular or high-risk multidisciplinary procedures, including angiography, embolization, carotid stenting, and advanced cardiopulmonary support. Beginning in 2022, utilization expanded substantially, driven primarily by intraoperative computed tomography, lesion localization, navigation support, and immediate procedural verification.

The largest annual increase occurred in 2023, when the cumulative number of procedures rose from 13 to 43. This transition reflected a shift from selected rescue or vascular-protection applications toward more routine incorporation of intraoperative imaging into operative planning, localization, and confirmation of procedural results.

### 3.3. Principal Clinical Applications and Multidisciplinary Involvement

Computed tomography localization or navigation remained the most common principal application, accounting for 41 procedures (74.5%). Representative uses included endoscopic sinus surgery, localization of deep-neck lesions, identification of teeth, fixation plates, screws, and other difficult anatomical targets, and support for operative planning. Immediate procedural verification was the principal application in 2 procedures (3.6%) and was used to confirm lesion removal, hardware removal, or completion of the intended anatomical result.

Angiography or endovascular intervention was the principal application in 11 procedures (20.0%). Of these, nine were planned preoperatively and performed as an integrated component of the same operative session for vascular assessment, embolization, carotid protection, or stent placement. Two procedures were performed as unplanned emergency/rescue interventions, including one for bleeding requiring transarterial embolization and one requiring urgent stent placement. These procedures included selective angiography, embolization, carotid stenting, covered-stent placement, bleeding control, and vascular protection. One procedure (1.8%) required advanced cardiopulmonary support with extracorporeal membrane oxygenation during high-risk thyroid and airway surgery.

Multidisciplinary participation included cardiovascular surgery in 11 procedures (20.0%), interventional radiology in 11 (20.0%), plastic and reconstructive surgery in 3 (5.5%), neurosurgery in 1 (1.8%), and thoracic surgery or the extracorporeal membrane oxygenation team in 1 (1.8%). Because multiple departments could participate in the same procedure, departmental involvement was not mutually exclusive. The distribution of principal applications and multidisciplinary contributions is presented in Table 2.

### 3.4. Perioperative Characteristics and Resource Utilization

Perioperative characteristics varied according to the hybrid operating room modality used (Table 3). These modality-based data are presented descriptively because the subgroups represented different clinical indications, baseline risks, and levels of procedural complexity. Robot-assisted procedures had a median anesthesia duration of 580 min (interquartile range, 450–612 min), a median operative duration of 335 min (250–368 min), and a median estimated blood loss of 115 mL (40–188 mL). Three of the four robot-assisted procedures (75.0%) required postoperative intensive care unit admission, and the median hospital length of stay was 12 days (7–19 days).

Procedures involving angiography or endovascular intervention had a median anesthesia duration of 468 min (269–571 min), a median operative duration of 300 min (174–335 min), and a median estimated blood loss of 60 mL (28–205 mL). All 11 patients in this subgroup were admitted to the intensive care unit postoperatively. The median intensive care unit stay was 2 days (2–4 days), and the median hospital length of stay was 12 days (9–22 days). Postoperative intubation was required in 7 patients (63.6%), and nasogastric tube use was documented in 5 (45.5%).

Procedures involving intraoperative computed tomography had a median anesthesia duration of 143 min (113–177 min), a median operative duration of 64 min (46–102 min), and a median estimated blood loss of 20 mL (10–50 mL). Two of 42 patients (4.8%) required postoperative intensive care unit admission, and the median hospital length of stay was 3 days (3–5 days).

The single extracorporeal membrane oxygenation-supported procedure required 480 min of anesthesia and 500 min of operative time, with an estimated blood loss of 65 mL. The patient required 12 days of intensive care, 4 days of postoperative intubation, 4 days of nasogastric tube use, and a total hospital stay of 16 days.

Across the entire cohort, the median anesthesia duration was 168 min (118–218 min), the median operative duration was 85 min (50–122 min), and the median estimated blood loss was 25 mL (10–72 mL). Thirteen patients (23.6%) required postoperative intensive care unit admission, 8 (14.5%) required postoperative intubation, and 8 (14.5%) required nasogastric tube placement. The median hospital length of stay was 3 days (3–6 days).

## 4. Discussion

### 4.1. Principal Findings

This seven-year single-center study describes the institutional patterns of hybrid operating room utilization in otolaryngology. Unlike previous procedure-specific reports, the present study characterizes longitudinal institutional utilization across multiple otolaryngology indications and hybrid operating room capabilities. Among 55 eligible procedures, computed tomography-based localization or navigation was the predominant principal application, accounting for approximately three-quarters of the cohort. Angiography or endovascular intervention represented the second major application, while approximately one-quarter of procedures involved direct participation by specialties other than otolaryngology–head and neck surgery.

The findings suggest two principal patterns of use. The first comprised image-guided localization, navigation, and procedural verification, particularly in sinonasal, nasopharyngeal, skull-base, deep-neck, oral, and maxillofacial procedures. The second comprised selected vascular and high-acuity procedures requiring angiography, endovascular intervention, or advanced cardiopulmonary support. The differences in anesthesia duration, intensive care utilization, and hospitalization across these groups should be interpreted primarily as reflecting differences in case selection and clinical complexity rather than comparative effects of the technologies used.

### 4.2. Evolution from High-Risk Vascular Procedures to Broader Image-Guided Applications

The temporal pattern observed in this cohort suggests an evolution in the institutional role of the hybrid operating room. Initial use was concentrated in complex vascular and high-risk multidisciplinary procedures, including embolization, carotid stenting, vascular protection, and extracorporeal membrane oxygenation-supported surgery. From 2022 onward, use expanded to a broader range of otolaryngology procedures, particularly those involving intraoperative computed tomography, lesion localization, navigation support, and immediate procedural verification [1].

This evolution reflects a transition from selective use in rescue or high-acuity situations toward more routine incorporation of intraoperative imaging into surgical planning and execution. The hybrid operating room therefore functioned not only as a setting for vascular intervention but also as an intraoperative diagnostic environment in which anatomical information could be acquired, interpreted, and applied during the same anesthetic session. Similar transitions toward integrated diagnostic and therapeutic workflows have been reported in other hybrid operating room settings [5,6,9,10].

### 4.3. Intraoperative Computed Tomography, Localization, and Procedural Verification

Intraoperative computed tomography was used primarily for anatomical localization, navigation, and confirmation of the intended surgical result. The relevance to otolaryngology is particularly evident in the complex three-dimensional anatomy of the sinonasal cavity and skull base, the proximity of the orbit, cranial nerves, and major vessels, and the restricted field of view encountered during endoscopic procedures. Altered anatomy, limited exposure, and previous treatment may further complicate conventional surgical orientation [2,3,11]. Cone-beam computed tomography provides detailed three-dimensional assessment of complex sinonasal anatomy and may complement intraoperative navigation in selected procedures [2].

The broader utility of intraoperative cone-beam computed tomography and navigation for lesion localization, procedural guidance, and immediate verification has also been demonstrated in thoracic, urological, and orthopedic procedures [12,13,14,15,16,17]. In the present otolaryngology cohort, updated three-dimensional imaging was used to localize deep lesions, teeth, fixation plates, screws, and other difficult anatomical targets [18,19]. Repeat imaging after the principal intervention also enabled immediate confirmation of lesion or hardware removal and completion of the intended anatomical result.

Procedures involving intraoperative computed tomography showed different perioperative profiles from those involving vascular or advanced support capabilities, including differences in operative and anesthesia duration, estimated blood loss, and hospitalization. These differences most likely reflect case selection, baseline risk, and procedural complexity and should not be interpreted as comparative effects of the technologies used.

### 4.4. Angiography, Endovascular Intervention, and High-Acuity Surgery

An important feature of the hybrid operating room is the ability to combine surgical and endovascular treatment without transferring an anesthetized patient to a separate interventional suite. In the present cohort, angiography and endovascular intervention were used in approximately one-fifth of procedures for indications including bleeding-source identification, embolization, carotid stenting, covered-stent placement, and vascular protection. Most endovascular procedures were planned as integrated components of the operative session, whereas two cases demonstrated the rescue capability of the hybrid operating room for unexpected vascular emergencies. This integrated workspace is highly relevant for tumors demanding immediate devascularization before excision, such as advanced juvenile nasopharyngeal angiofibroma [4].

Immediate access to angiography and endovascular treatment provided an additional management option during complex surgery. Streamlined transitioning between open or endoscopic surgical resection and endovascular intervention without relocating the patient offers an important operational advantage by avoiding the need to transfer an anesthetized or clinically unstable patient between procedural areas [20]. Furthermore, advanced intraoperative navigation combined with immediate angiographic capabilities provides an additional option for vascular assessment and management when unexpected hemorrhage or vascular injury occurs within the intricate confines of the skull base [21]. Similar hybrid workflows have been used in neurosurgical, trauma, and vascular settings to provide immediate angiographic assessment and endovascular treatment without transferring the patient between procedural areas [22,23,24,25,26,27,28].

The single extracorporeal membrane oxygenation-supported procedure further illustrates the potential role of the hybrid operating room in patients with severe airway compromise or anticipated cardiopulmonary instability [29,30,31,32]. Combining open cervicothoracic access with continuous physiological support mirrors complex trauma configurations, where bedside angiographic assessments and ECMO stabilization are handled simultaneously within a unified surgical field. Because this application was represented by only one case, it should be considered an illustrative high-acuity use rather than evidence of a generalizable outcome benefit.

### 4.5. Multidisciplinary Integration and Clinical Implications

The hybrid operating room provided a common procedural environment for otolaryngology–head and neck surgery, cardiovascular surgery, interventional radiology, plastic and reconstructive surgery, neurosurgery, and thoracic surgery. Feasibility data utilizing monoplane robotic C-arm systems highlight the optimization of multi-specialty workflows for endovascular access and cross-disciplinary interventions within shared hybrid rooms [6,24]. In our study, cardiovascular surgery and interventional radiology were the most frequently involved specialties, principally contributing to angiography, embolization, carotid stenting, covered-stent placement, and vascular protection. Plastic and reconstructive surgery participated in reconstruction after complex oncologic or cervical procedures, while neurosurgical and thoracic teams supported selected skull-base and extracorporeal membrane oxygenation-assisted cases.

The value of this multidisciplinary model lies in the ability to coordinate imaging, surgical resection, vascular intervention, reconstruction, and advanced physiologic support within a single operative episode. The physical convergence of traditional surgical equipment and advanced interventional radiology systems demands a high level of coordination among diverse specialists, which requires standardized communication, clearly defined responsibilities, and consistent adherence to safety protocols [33]. Furthermore, complex skull-base surgeries and salvage procedures for unexpected vascular injuries may benefit from coordinated access to surgical and endovascular expertise [34]. Such integration reduces delays between diagnostic assessment and intervention and avoids transport of unstable or anesthetized patients [9,21].

Nevertheless, successful implementation requires coordinated scheduling, clearly defined team roles, familiarity with imaging and radiation-safety protocols, and institutional investment in specialized equipment and personnel. Radiation protection should follow the principle of as low as reasonably achievable through dose optimization, appropriate collimation, minimization of repeated imaging and fluoroscopy, adequate shielding, personal dosimetry, and staff training. Attention should also be given to cumulative occupational exposure among personnel who regularly participate in hybrid operating room procedures [34,35].

Cost is another important consideration in hybrid operating room implementation. These facilities require substantial capital investment and ongoing equipment, maintenance, and personnel costs. Published economic evaluations suggest that cost-effectiveness depends on procedural volume, case selection, institutional workflow, and the potential to avoid patient transfer or additional procedures, with favorable findings reported in selected vascular and trauma settings [36,37]. However, these findings are context-specific, and the cost-effectiveness of hybrid operating room utilization in otolaryngology remains uncertain.

From a practical perspective, the present findings suggest that hybrid operating room utilization in otolaryngology may be organized around two broad clinical pathways. The first involves image-guided localization, navigation, and procedural verification in anatomically complex but generally lower-acuity procedures. The second involves vascular or cardiopulmonary support for high-risk oncologic, skull base, airway, or hemorrhagic conditions. Recognizing these distinct pathways may assist institutions in case selection, staffing, equipment planning, and the development of multidisciplinary protocols.

### 4.6. Integration with Robot-Assisted Surgery

Robot-assisted surgery represented another complementary use of the hybrid operating room in this cohort. Four procedures incorporated a robot-assisted approach, primarily for complex head and neck or deep-space lesions in which restricted access, altered anatomy, or proximity to major vascular structures increased procedural difficulty. The hybrid environment supplemented the enhanced visualization and instrument dexterity of the robot-assisted platform by providing intraoperative computed tomography localization, immediate anatomical assessment, and, when necessary, coordinated vascular intervention. Similar integration of robotic systems with intraoperative imaging and navigation has been described in other surgical disciplines, supporting the broader feasibility of image-guided robotic procedures within the hybrid operating room environment [38,39]. However, given the small and heterogeneous subgroup, these findings support feasibility rather than comparative benefit.

### 4.7. Limitations

This study has several limitations. First, its retrospective design relied on the accuracy and completeness of electronic medical records, operative reports, imaging records, and procedure codes. Some procedures may have been missed if hybrid operating room use was incompletely documented, while the clinical purpose or timing of imaging could not always be reconstructed with certainty.

Second, selection for the hybrid operating room was based on individualized preoperative clinical and technical assessment rather than uniform allocation criteria throughout the study period. Decisions reflected the anticipated need for intraoperative imaging or navigation, procedural verification, endovascular intervention, or advanced cardiopulmonary support, with multidisciplinary consultation when indicated. Selection bias related to case complexity, clinical judgment, and evolving institutional practice therefore cannot be excluded.

Third, this was a single-center study conducted in an institution with a specific combination of imaging systems, surgical expertise, and multidisciplinary resources. The findings may not be directly generalizable to centers with different equipment, referral patterns, staffing models, or thresholds for hybrid operating room use.

Fourth, the cohort was clinically heterogeneous, ranging from image-guided localization procedures to complex vascular, robot-assisted, and extracorporeal membrane oxygenation-supported procedures. This heterogeneity limits the interpretability of pooled perioperative data and precludes direct comparison among procedural groups. In addition, some modality-based subgroups were not mutually exclusive because individual procedures could incorporate more than one hybrid operating room capability.

Fifth, the study did not include a conventional operating room control group. It therefore cannot determine whether hybrid operating room use reduced complications, operative time, blood loss, transport-related risk, reoperation, or cost. Patient radiation dose, occupational exposure, cumulative staff exposure, and the impact of intraoperative imaging on real-time surgical decision-making were not systematically documented. Long-term functional, oncologic, and quality-of-life outcomes were not assessed.

Finally, several early complex cases had been described previously in a brief report [1]. These cases were retained to provide a complete longitudinal account of institutional utilization, were identified as previously reported, and were counted only once. The present analysis addresses a broader and distinct research question focused on temporal evolution, clinical applications, multidisciplinary integration, and perioperative characteristics. Future prospective multicenter studies should define standardized indications, record whether intraoperative imaging changes surgical management, compare hybrid and conventional operating room workflows, and evaluate radiation exposure, resource utilization, costs, complications, and long-term clinical outcomes.

## 5. Conclusions

The hybrid operating room has evolved into an integrated procedural platform for otolaryngology, extending from selected vascular and high-acuity interventions to broader applications in intraoperative imaging, anatomical localization, navigation, and procedural verification. In this single-center cohort, computed tomography-based applications predominated, particularly for procedures involving the sinonasal cavity, nasopharynx, and skull base, whereas angiographic, endovascular, and cardiopulmonary support capabilities were used during the management of more complex cases requiring multidisciplinary participation. The observed differences in perioperative resource utilization primarily reflected variation in case complexity and clinical severity rather than comparative effectiveness among modalities. Further prospective multicenter studies are needed to establish standardized indications and evaluate safety, complications, radiation exposure, workflow efficiency, and long-term clinical outcomes.

## Figures and Tables

**Table 1:** Patient and procedural characteristics of the otolaryngology hybrid operating room study cohort.

Characteristic	Overall Cohort (*n* = 55)
Age (IQR), years	46.0 (33.0–58.5)
Age range, years	5–76
**Sex**	
Male	33 (60.0)
Female	22 (40.0)
**Primary anatomical site**	
Sinonasal cavity, nasopharynx, or skull base	33 (60.0)
Neck or thyroid	10 (18.2)
Oral cavity or mandible	5 (9.1)
Oropharynx or larynx	4 (7.3)
Salivary gland	3 (5.5)
Robot-assisted procedures	4 (7.3)
Documented multidisciplinary procedures	13 (23.6)
**Comorbidities documented in available records (*n* = 47)**	
Hypertension	9 (19.1)
Diabetes mellitus	4 (8.5)
Cardiovascular or cerebrovascular disease	2 (4.3)
Hyperlipidemia	2 (4.3)
Gout	3 (6.4)
Chronic hepatitis	1 (2.1)
Asthma	1 (2.1)

**Table 2:** Principal clinical applications and multidisciplinary involvement in hybrid operating room procedures.

(**A**) Principal clinical applications			
**Principal Application**	**No. of Procedures (%)**	**Representative Procedures**	**Principal Reason for Hybrid Operating Room Use**
CT localization/navigation	41 (74.5)	Endoscopic sinus surgery; deep-neck, dental, or hardware localization	Anatomical localization, navigation support, and operative planning
Immediate procedural verification	2 (3.6)	Post-resection CT; confirmation after lesion or hardware removal	Confirmation of lesion removal, anatomical result, or completion of the intended procedure
Angiography/endovascular intervention	11 (20.0)	Angiography; embolization; carotid or covered-stent placement	Vascular assessment, bleeding control, carotid protection, or stent placement
Advanced cardiopulmonary support	1 (1.8)	ECMO-supported thyroid surgery	Airway and cardiopulmonary protection during high-risk surgery
(**B**) Multidisciplinary departmental involvement			
**Department**		**No. of Procedures (%)**	**Principal Contribution**
Cardiovascular Surgery		11 (20.0)	Carotid stenting, covered-stent placement, angiography, and vascular protection
Interventional Radiology		11 (20.0)	Angiographic assessment, identification of bleeding sources, and embolization
Plastic and Reconstructive Surgery		3 (5.5)	Reconstruction following complex oncologic or cervical surgery
Neurosurgery		1 (1.8)	Management of a skull-base complication involving carotid injury
Thoracic Surgery/ECMO team		1 (1.8)	Extracorporeal support for severe airway compromise

Departments were not mutually exclusive because more than one specialty could participate in the same procedure. Abbreviations: CT, computed tomography; ECMO, extracorporeal membrane oxygenation.

**Table 3:** Perioperative characteristics and short-term outcomes according to hybrid operating room modality.

Characteristic	Robot-Assisted Procedures (*n* = 4)	Angiography/ Endovascular Intervention (*n* = 11)	Intraoperative CT Procedures (*n* = 42)	ECMO-Supported Procedure (*n* = 1)	Overall Cohort (*n* = 55)
**Operative variables**					
Anesthesia duration, min	580 (450–612)	468 (269–571)	143 (113–177)	480	168 (118–218)
Operative duration, min	335 (250–368)	300 (174–335)	64 (46–102)	500	85 (50–122)
Estimated blood loss, mL	115 (40–188)	60 (28–205)	20 (10–50)	65	25 (10–72)
**Postoperative care**					
Postoperative intensive care unit admission	3 (75.0)	11 (100.0)	2 (4.8)	1 (100.0)	13 (23.6)
Intensive care unit length of stay, days	2 (1–2)	2 (2–4)	0 (0–0)	12	0 (0–0)
Postoperative intubation	2 (50.0)	7 (63.6)	0 (0.0)	1 (100.0)	8 (14.5)
Postoperative intubation duration, days	1 (0–2)	1 (0–2)	0 (0–0)	4	0 (0–0)
Nasogastric tube use	1 (25.0)	5 (45.5)	3 (7.1)	1 (100.0)	8 (14.5)
Nasogastric tube duration, days	0 (0–0)	0 (0–3)	0 (0–0)	4	0 (0–0)
**Hospital outcome**					
Hospital length of stay, days	12 (7–19)	12 (9–22)	3 (3–5)	16	3 (3–6)

Note: Data are presented as median (interquartile range) or number (percentage). Modality-based subgroups may overlap; therefore, subgroup counts should not be summed. The ECMO-supported procedure is presented descriptively, and no between-group comparisons were performed.

## Data Availability

The de-identified raw spreadsheets supporting the findings of this study are publicly available at https://dataverse.harvard.edu/dataset.xhtml?persistentId=doi:10.7910/DVN/KQPIEM (accessed on 1 June 2026). The final analytic cohort was derived from these files after eligibility verification, duplicate removal, and application of the exclusion criteria described in the Section 2.

## References

[1] Tsai J.H., Chang C.H., Lee C.H., Lin F.C., Tsai S.C. (2022). Innovative applications of hybrid operating room in otolaryngology: A pilot study. Oral Oncol..

[2] Han M., Kim H.J., Choi J.W., Park D.Y., Han J.G. (2022). Diagnostic usefulness of cone-beam computed tomography versus multi-detector computed tomography for sinonasal structure evaluation. Laryngoscope Investig. Otolaryngol..

[3] Ferrari M., Daly M.J., Douglas C.M., Chan H.H.L., Qiu J., Deganello A., Taboni S., Thomas C.M., Sahovaler A., Jethwa A.R. (2019). Navigation-guided osteotomies improve margin delineation in tumors involving the sinonasal area: A preclinical study. Oral Oncol..

[4] He Y., Hong R., Wang S., Wu J., Li W., Zhang H., Xue K., Liu Q., Gu Y., Sun X. (2025). Preoperative Embolization Followed by Tumor Resection Without Time Interval in Advanced Juvenile Nasopharyngeal Angiofibroma. Cardiovasc. Interv. Radiol..

[5] Jiao Y., Wang P., Wang M., Wang S., Zhao J., Cao Y. (2025). Salvage endovascular treatment in hybrid operating room for incidental internal carotid artery injury during neurosurgery: A single-center experience. Neurosurg. Rev..

[6] Ferdowssian K., Pigorsch M., Kiss I., Morsi K., Ďuriš M., Sila D., Naghibi-Saber S.A., Tilgner J., Moszko S., Ciolpan M. (2026). Neuroendovascular procedures in the hybrid operating room using a monoplane robotic C-arm-feasibility study. Clin. Neurol. Neurosurg..

[7] Chang L.K., Su P.K., Malwade S., Chung W.Y., Chan P.S., Chen S.C., Chen L.C., Yang S.M. (2025). Enhancing Microwave Ablation for Lung Lesions with Cone-Beam Computed Tomography Guidance and Intrapulmonary Fine Adjustment in a Hybrid Operating Room. Acad. Radiol..

[8] Zemela M.S., Tjaden B.L. (2025). Angiographic evaluation of lower extremity acute limb ischemia in an extracorporeal membrane oxygenation patient using bedside portable radiograph. J. Vasc. Surg. Cases Innov. Tech..

[9] Gómez-Amador J.L., Rodriguez-Hernandez L.A., Aragón-Arreola J.F., Mondragón-Soto M.G., Munuzuri-Camacho M.A., Moncada-Habib J.T., Domínguez-García A., Guinto-Nishimura G.Y., Villalobos-Díaz R. (2026). Simultaneous endoscopic endonasal treatment of a GH-secreting PitNET and paraclinoid aneurysm in a hybrid operating room-2D operative video. Surg. Neurol. Int..

[10] Gharios M., El-Hajj V.G., Frisk H., Ohlsson M., Omar A., Edström E., Elmi-Terander A. (2023). The use of hybrid operating rooms in neurosurgery, advantages, disadvantages, and future perspectives: A systematic review. Acta Neurochir..

[11] Valtonen O., Bizaki A., Kivekäs I., Rautiainen M. (2018). Three-Dimensional Volumetric Evaluation of the Maxillary Sinuses in Chronic Rhinosinusitis Surgery. Ann. Otol. Rhinol. Laryngol..

[12] Chen L.C., Su P.K., Hu G.N., Malwade S., Chung W.Y., Chang L.K., Yang S.M. (2026). Cone-beam computed tomography laser-guided transthoracic needle biopsy for pulmonary lesions in a hybrid operating room: Feasibility study by an interventional pulmonologist. Diagnostics.

[13] Mammana M., Zambello G., Busetto A., Cataldi G., Zaraca F., Dell’Amore A. (2026). Step-by-step lung nodule localization in the hybrid operating room using a double marking technique with Lipiodol and indocyanine green. Multimed. Man. Cardiothorac. Surg..

[14] Roemeling S., Kingma R.A., Suijker C.A., Altobelli E., Bus M.T.J., Greuter M.J.W., Mahesh S.V.K., de Jong I.J. (2025). Intraoperative Cone Beam Computed Tomography Increases Single Procedure Stone-Free Rates in Percutaneous Nephrolithotomy: Results of a Randomized Controlled Trial. J. Endourol..

[15] Suijker C.A., Kingma R.A., van Oort I.M., Roemeling S. (2026). Exploratory Treatment-Selection Model of Intraoperative Cone-Beam Computed Tomography During Percutaneous Nephrolithotomy: Insights from RCT Data. J. Clin. Med..

[16] Sola N.M., van de Wall B.J.M., Haefeli P.C., Beeres F.J.P., Babst R., Link B.C., Haveman R.A. (2025). The use of 3D computer-assisted navigation and its influence on radiation exposure and operation time in the surgical treatment of fragility fractures of the pelvis. Injury.

[17] Haida D.M., Meiswinkel F., Prosenikov G., Huber-Wagner S. (2026). Retrograde posterior column acetabular screw: Robotically assisted and CT-guided minimally invasive procedure. Unfallchirurgie.

[18] Valencia-Sanchez B.A., Hsue V.B., Leuin S.C., Patel V.A. (2025). Endoscopic Retrieval of Displaced Maxillary Third Molar Crown: A Pediatric Endonasal Approach. Ear Nose Throat J..

[19] Lim D., Parumo R., Chai M.B., Shanmuganathan J. (2017). Transnasal Endoscopy Removal of Dislodged Dental Implant: A Case Report. J. Oral Implantol..

[20] Baig O., Snyder S.J., Yu N.S., Gandhi O.H., Almasri S., Choudhri O.A. (2026). Use of Advanced Intraoperative Navigation for Percutaneous Transorbital Inferior Ophthalmic Vein Access in Embolization of a Residual Type D Carotid-Cavernous Fistula. World Neurosurg..

[21] Pavesi G., Rechberger J.S., Millesi E., Cavallo S.M., Serpico F., Valluzzi A., Vallone S., Iaccarino C., Dimitriadis S. (2026). Combined One-Step Hybrid Treatment for a Pediatric Giant Internal Carotid Artery Aneurysm: A Case Report. J. Neurol. Surg. A Cent. Eur. Neurosurg..

[22] Pitoulias A.G., Dimitrios K., Taneva G.T., Donas K.P. (2026). Advantages of implementing a hybrid operating room for endovascular aortic aneurysm repair at a new vascular surgery centre. Vasa.

[23] Bowman K.M., Dawkins D., Schaefer S., Scheuermann J., Sulaimanov U., Ahmed A.S., Niemann D., Baskaya M.K., Aagaard-Kienitz B. (2026). Intravenous 3-Dimensional Digital Subtraction Angiography: A Noninvasive, High-Quality Alternative to Cerebral Catheter Angiography. Oper. Neurosurg..

[24] Cabot J.H., Zubkov M., Knowlton L.M., Romagnoli A., Kauvar D.S., PROspective Observational Vascular Injury Treatment Study Investigators (2025). Hospital setting of endovascular repair influences procedural outcomes in blunt traumatic aortic injury. J. Vasc. Surg..

[25] Colistra D., Giordano M., Boeris D., Cenzato M. (2025). Selective intra-arterial indocyanine green video angiography in a hybrid operating theatre for a ruptured arteriovenous malformation involving Broca’s area. BMJ Case Rep..

[26] Ozcinar E., Akca F., Saricaoglu M.C., Hasde A.I., Dikmen N., Buyukcakir O., Guven A., Durmaz O., Boga S.A., Karacuha A.F. (2025). Total Endovascular Aortic Arch Repair Using In Situ Needle Triple Fenestration and Selective Cerebral Perfusion: Single-Center Results. J. Clin. Med..

[27] Yürekli İ., İner H., Yazman S., Gökalp O., Çakır H., Yılık L., Gürbüz A. (2025). Outcomes of endovascular aortic aneurysm repair in a long-term real-world cohort: Analysis from the first hybrid operating room in Türkiye. Turk. Gogus Kalp Damar Cerrahisi Derg..

[28] Zhao L., Liu J., Qiao Z., Qiu Y., Zhou Y., Wang J. (2025). Multimodal image fusion guidance for the treatment of structural heart disease and aortic dissection in hybrid operation room: A single-center case series in a hybrid operating room. Quant. Imaging Med. Surg..

[29] Wu H., Zhuo K., Cheng D. (2023). Extracorporeal membrane oxygenation in critical airway interventional therapy: A review. Front. Oncol..

[30] Lin J., Frye L. (2021). The intersection of bronchoscopy and extracorporeal membrane oxygenation. J. Thorac. Dis..

[31] Garcia S.I., Smischney N.J., Sandefur B.J., D’Andria Ursoleo J., Kelm D.J., Wieruszewski P.M. (2025). Peri-intubation cardiovascular collapse during emergency airway management. Pulm. Ther..

[32] Su S., Liang L., Liu Z., Wang L., Zhang T., Chen N. (2025). Extracorporeal membrane oxygenation as a life-saving bridge for patients with airway obstruction caused by neck and chest tumors to salvage procedures: An in-depth review. Int. J. Surg..

[33] Rhodin J., Erestam S., Bazzi M., Milton J., Strömberg S., Pettersson M. (2026). The Influence of Age and Experience on Safety Climate Perceptions Among Healthcare Staff in Operating, Interventional Radiology, and Hybrid Operating Rooms: A Cross-Sectional Study. J. Multidiscip. Healthc..

[34] Rennie N., Soenens G., Meyns I., Legein Z., Vlerick P., Vanpeteghem C., Bruneel B., Van Herzeele I. (2026). Mind the Gap: Reported Versus Observed Surgical Safety Checklist Time-Out Adherence in the Hybrid Operating Room. World J. Surg..

[35] Soenens G., Vlerick P., Bacher K., Rennie N., Grantcharov T., Van Herzeele I. (2025). Impact of a Massive Open Online Course on Radiation Safety in the Hybrid Operating Room. Eur. J. Vasc. Endovasc. Surg..

[36] Patel S., Lindenberg M., Rovers M.M., van Harten W.H., Ruers T.J.M., Poot L., Retel V.P., Grutters J.P.C. (2022). Understanding the costs of surgery: A bottom-up cost analysis of both a hybrid operating room and conventional operating room. Int. J. Health Policy Manag..

[37] Kinoshita T., Moriwaki K., Hanaki N., Kitamura T., Yamakawa K., Fukuda T., Hunink M.G.M., Fujimi S. (2021). Cost-effectiveness of a hybrid emergency room system for severe trauma: A health technology assessment from the perspective of the third-party payer in Japan. World J. Emerg. Surg..

[38] Balicki M., Kyne S., Toporek G., Holthuizen R., Homan R., Popovic A., Burström G., Persson O., Edström E., Elmi-Terander A. (2020). Design and control of an image-guided robot for spine surgery in a hybrid OR. Int. J. Med. Robot..

[39] Gao X., Chao Y.K. (2022). Thoracic surgery in Taiwan. J. Thorac. Dis..

